# Patient and caregiver benefit‐risk preferences for nonmetastatic castration‐resistant prostate cancer treatment

**DOI:** 10.1002/cam4.3321

**Published:** 2020-07-29

**Authors:** Sandy Srinivas, Ateesha F. Mohamed, Sreevalsa Appukkuttan, Marc Botteman, Xinyi Ng, Namita Joshi, Jui‐Hua Tsai, Jarjieh Fang, A. Reginald Waldeck, Stacey J. Simmons

**Affiliations:** ^1^ Stanford University Medical Center Palo Alto CA USA; ^2^ Bayer U.S. LLC Whippany NJ USA; ^3^ Pharmerit International, LP Bethesda MD USA

**Keywords:** Caregivers, Choice Behavior, Patients, Prostatic Neoplasms, Castration‐Resistant, Risk Assessment

## Abstract

**Background:**

Recently approved second‐generation androgen receptor inhibitors (SGARIs) for non‐metastatic castration‐resistant prostate cancer (nmCRPC) have similar efficacy but differ in safety profiles. We used a discrete choice experiment (DCE) to examine how nmCRPC patients and caregivers perceive the benefits versus risks of these new treatments.

**Methods:**

An online DCE survey with 14 treatment choice questions was administered to nmCRPC patients and caregivers. Each choice question compared two hypothetical medication profiles varying in terms of 5 safety attributes (risk or severity of adverse events [AEs]: fatigue, skin rash, cognitive problems, serious fall, and serious fracture) and two efficacy attributes (duration of overall survival [OS] and time to pain progression). Random parameters logit models were used to estimate each attribute's relative importance. We also estimated the amounts of OS that respondents were willing to forego for a reduction in AEs.

**Results:**

In total, 143 nmCRPC patients and 149 caregivers viewed the AEs in following order of importance (most to least): serious fracture, serious fall, cognitive problems, fatigue, and skin rash. On average, patients were willing to trade 5.8 and 4.0 months of OS to reduce the risk of serious fracture and fall, respectively, from 3% to 0%; caregivers were willing to trade 6.6 and 5.4 months of OS.

**Conclusions:**

nmCRPC patients and caregivers preferred treatments with lower AE burdens and were willing to forego OS to reduce the risk and severity of AEs. Our results highlight the importance of carefully balancing risks and benefits when selecting treatments in this relatively asymptomatic population.

## Graphical abstract

Nonmetastatic castration‐resistant prostate cancer patients and caregivers preferred treatments with lower adverse event (AE) burden and were willing to forego survival to reduce the risks and severity of AEs. They viewed AEs in following order of importance (most to least): serious fracture, serious fall, cognitive problems, fatigue, and skin rash. It is important to carefully balance the risks and benefits when selecting treatments in this relatively asymptomatic population, especially given the differing AE profiles of newer treatments.

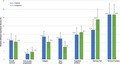

## 1 INTRODUCTION

Prostate cancer is one of the most common cancers affecting men, with an estimated 174 650 new cases and 31 620 deaths in 2019 in the United States (US).^1^ Most patients on androgen deprivation therapy (ADT) eventually become castration‐resistant, meaning they progress with biochemical recurrence with rising prostate‐specific antigen (PSA) levels despite castrate levels of testoterone.^2^ Uncontrolled rising PSA levels have been shown to result in anxiety in patients.^3^ Progression to the metastatic state is associated with mortality and contributes to a substantial proportion of prostate cancer deaths.^2^, ^4^, ^5^ Therefore, non‐metastatic castration‐resistant prostate cancer (nmCRPC) is a critical period during which therapeutic interventions can delay prostate cancer progression to the metastatic state. Until recently, nmCRPC was most commonly managed with active surveillance or continued ADT with first generation androgen receptor (AR) antagonists.^2^, ^6^ A recent real‐world study conducted in the US using the 2015–2017 Ipsos Global Oncology Monitor Database observed that the most common treatments used in nmCRPC during that period were luteinizing hormone‐releasing hormone agonists and antiandrogens.^7^ The use of first generation androgen inhibitors has not been shown to yield significant survival benefits in nmCRPC.^2^

Since 2018, the treatment options for nmCRPC have expanded with the approval of several second‐generation androgen receptor inhibitors (SGARIs) in the US.^8^, ^9^, ^10^ Large phase 3 trials demonstrated that these SGARIs provide significant benefits in prolonging metastasis‐free survival (MFS) among men with nmCRPC, with median MFS ranging from 36.6 to 40.5 months across all 3 trials.^11^, ^12^, ^13^ More recently, data demonstrating improved overall survival (OS) with SGARIs therapy have emerged, where the newly‐approved SGARIs were shown to be associated with a 25% to 31% reduction in the risk of death.^14^, ^15^, ^16^ Compared to the first generation antiandrogens, SGARIs also have increased specificity, higher affinity to the androgen receptor, and are not associated with androgen withdrawal syndrome.^17^ As such, SGARIs have the potential to become the new standard of care.

However, trial results also suggest that SGARIs have different safety profiles, even after adjusting for cross‐trial heterogeneity.^18^ For example, the reported rates of fatigue, the most common adverse event (AE) in these trials, ranged from 12% to 33%.^11^, ^12^, ^13^ Rates of central nervous system related AEs vary among the SGARIs due to different penetration of the blood‐brain barrier.^19^ Given that nmCRPC is fairly asymptomatic, an important treatment goal is to minimize AEs, as they can interfere with patients’ daily activities, affecting quality of life (QoL), and may lead to treatment discontinuation.^20^ Hence, careful weighing of the efficacy and AEs of treatment is crucial in nmCRPC treatment decision‐making. It has been shown that the majority of men with prostate cancer desired an active or collaborative role in treatment decision‐making.^21^ Several studies have examined treatment preferences of prostate cancer patients and caregivers,^22^, ^23^, ^24^, ^25^, ^26^, ^27^ but they were not specific to the new nmCRPC treatment landscape. There remains a need to understand the importance that nmCRPC patients and caregivers attribute to avoiding AEs unique to the new SGARI therapies, and how these AEs may influence their treatment choices in a disease state that is asymptomatic.

Discrete choice experiments (DCEs) are commonly employed to quantify benefit‐risk preferences for healthcare interventions.^28^ In a DCE, respondents choose between sets of alternative hypothetical treatment profiles with varying levels of treatment characteristics (ie attributes). Using this approach, while following best‐practice recommendations,^29^ we aimed to elicit the treatment preferences of nmCPRC patients and caregivers of such patients, and to examine the extent to which they are willing to forego gains in OS to avoid or minimize AEs.

## 2 MATERIALS AND METHODS

### 2.1 Study design and sample

**Figure 1:**
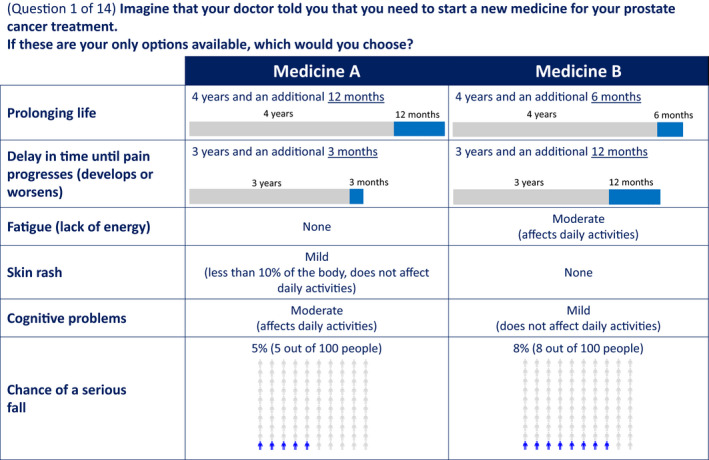
Example of Choice Question in the Discrete Choice Experiment. Risk of a serious fracture was shown in half of the choice tasks and risk of a serious fall was shown in the other half. For the caregiver DCE, the question reads *“Imagine that the doctor told you that the patient that you are caring for needs to start a new medicine for his prostate cancer treatment. If these are the only options available, which would you choose?”*

This was a non‐interventional cross‐sectional survey study of nmCRPC patients and caregivers that recruited and collected data via the administration of an online survey. The online survey included a DCE where respondents were presented with 14 choice questions (Figure 1) in which they selected a preferred option from sets of hypothetical medication profiles that varied systematically in attribute levels. The survey was administered online from February to April 2019 to a convenience sample of 150 patients and 150 caregivers recruited from online panels. Patients were self‐screened electronically using a screener adapted from a prior study.^30^ Eligibility was based on the following criteria: ≥18 years old, diagnosed with prostate cancer, had undergone surgical or medical castration, had rising PSA levels, and was not told by a physician that the prostate cancer had spread to any other part of his body. Caregivers were eligible if they were the primary caregiver of a patient who fulfilled the aforementioned criteria. Both patients and caregivers were excluded if they were unwilling to provide consent to participate in the study, or had taken part in a similar survey in the past 6 weeks. The electronic screener used is included in the online appendix. The study protocol was approved by a centralized US Institutional Review Board.

### 2.2 Survey development

The survey comprised two main sections: (1) questions assessing self‐reported demographic and disease‐related characteristics, and (2) DCE assessing benefit‐risk preferences. The DCE assessed two efficacy‐related attributes (OS and time to pain progression [TPP]) and five AE‐related attributes (frequency or severity of fatigue, skin rash, cognitive problems, risk of serious fall, and risk of serious fracture) (Table 1). These attributes were selected based on a targeted literature search of preference studies in prostate cancer and clinical trials of SGARIs,^11^, ^12^, ^13^, ^22^, ^23^, ^24^, ^25^, ^26^, ^27^ and individual phone interviews with five nmCRPC‐treating physicians, five nmCRPC patients, and five caregivers of nmCRPC patients. The interviews evaluated the relevance and saliency of the attributes from all perspectives. During the interviews, respondents were also asked to rate the importance of attributes and explain the rationale for their ratings. The five AE‐related attributes were selected for further analysis in the DCE because they were most commonly rated as highly important to the respondents and cited to potentially lead to serious consequences or can have a significant impact on daily activities. Additionally, the selection of AEs was also guided by what is frequently reported with SGARIs in literature.^31^, ^32^ The final five AEs selected also aligned with the AEs included in an independent review of the effectiveness and value of antiandrogens in nmCRPC by the Institute of Clinical and Economic Review.^33^ OS was ultimately selected because it was more clinically meaningful for patients who had difficulties drawing inference that MFS was a strong surrogate or predictor of OS as demonstrated in several analyses.^34^, ^35^ Furthermore, although MFS was used as an endpoint in trials, the definition varied. We also observed in our qualitative interviews that patients, caregivers, and physicians, all viewed OS as the most important efficacy attribute.

**Table 1:** Attributes, Attribute Labels, and Levels Included in the Discrete Choice Experiment

Attributes	Attribute Labels	Levels
Overall survival	Prolonging life	4 years and an additional 12 months 4 years and an additional 6 months 4 years and an additional 3 months
Time to pain progression	Delay in time until pain progresses (develops or worsens)	3 years and an additional 12 months 3 years and an additional 6 months 3 years and an additional 3 months
Fatigue	Fatigue (lack of energy)	None Mild (does not affect daily activities) Moderate (affects daily activities)
Skin rash	Skin rash	None Mild (less than 10% of the body, does not affect daily activities) Moderate (10%‐30% of the body, affects daily activities)
Cognitive problems	Cognitive problems	None Mild (does not affect daily activities) Moderate (affects daily activities)
Serious fall	Chance of a serious fall	None 5% (5 out of 100 people) 8% (8 out of 100 people)
Serious fracture	Chance of a serious fracture (broken bone)	None 5% (5 out of 100 people) 8% (8 out of 100 people)

A commonly used algorithm in SAS 9.4 was used to create the pairs of hypothetical medication profiles for the DCE.^36^ Four survey versions were created, each with 14 medication choice questions, and each patient or caregiver was randomized to one of the versions. Attribute descriptions (see online Tables S1 and S2) were included before the medication choice questions (profiles) to familiarize respondents with the attributes and levels. The descriptions for the AE attributes and their respective severity levels were adapted from the Common Terminology Criteria for Adverse Events (CTCAE). In general, a moderate AE affects the ability to maintain daily activities while a mild AE does not. For serious fall and serious fracture, we used risk grids to help respondents visualize the risk probabilities. Risk grids are commonly used for risk presentations in DCEs. Two risk‐grid questions were also included before the choice questions to illustrate the risk probabilities for serious fall and fracture, and to assess respondents’ understanding of the presented probabilities (see online appendix). A “dominance test” question was added whereby one medication profile was dominant over the other (ie higher levels of efficacy and lower levels of AEs) with the assumption that respondents should logically choose the dominant profile.

The initial draft survey was pretested via face‐to‐face interviews with five patients and five caregivers using the “think‐aloud” technique where respondents were asked to verbalize their thoughts while completing the survey. This assessed whether they comprehended the survey, and if they were willing to make trade‐offs amongst the attributes. During pretesting, it was observed that patients and caregivers were focused on avoiding severe AEs and were not willing to consider the benefits of extending OS if severe fatigue, skin rash, and cognitive problems were present. Therefore, to allow for trade‐offs, the severity levels were modified to none, mild, and moderate. Based on the feedback, the range of levels for the efficacy attributes and the risk probabilities for serious fall and serious fracture were slightly expanded.

### 2.3 Statistical analysis

Sociodemographic and disease‐related characteristics were summarized descriptively. Quality checks for DCE responses were evaluated and respondents were excluded from the analysis if they did not display variability in responses (ie chose either “medication A” only or “medication B” only across all 14 choice questions). Respondents who failed the dominance test question and completed the survey in less than 6.4 minutes (5th percentile of the distribution) were also excluded from the analysis.

Individual effects‐coded random parameter logit (RPL) models were used to model patients’ and caregivers’ choices in the DCE as a function of the attribute levels.^37^ The coefficients from the RPL represent the preference weights, which indicate the relative strength of preference for each attribute level. The difference in preference weights between an attribute's best and worst levels is also a measure of the overall relative importance of the attribute over the ranges measured in the DCE.^37^ Relative attribute importance scores (RAISs) were calculated by expressing the difference in preference weights as a percentage of the summation of the total differences across all attributes. The larger the RAIS, the greater the influence that a change in the attribute's levels has on treatment choices. Using the estimated preference weights, we calculated the rate at which patients and caregivers were willing to forego OS for a given reduction in AE risk or severity (ie the marginal rate of substitution [MRS]). In the MRS calculations, we specified OS as a continuous variable as this was supported by model fit statistics. All DCE analyses were using STATA/IC 14.2 and R Studio 3.5.0.

## 3 RESULTS

### 3.1 Sample characteristics

Of the 1466 adult patients with prostate cancer and 639 adult caregivers of patients with prostate cancer who accessed the survey link and initiated the electronic screening, 188 patients (12.8%) and 178 caregivers (27.9%) were eligible. Thirty‐eight patients and 28 caregivers did not complete the survey; of those who did complete it, 7 patients and 1 caregiver were excluded from the study analysis based on the quality checks. Therefore, the final sample included in the study analysis comprised 143 patients and 149 caregivers. A detailed screening flowchart is included in the online appendix.

Key sociodemographic and disease‐related characteristics of the patients and caregivers are summarized in Table 2. For the nmCRPC patient sample, the mean age was 53 years old (SD: 14.2) and the majority were white (84.6%), married (72.7%), had a college education or above (83.9%), and were employed full‐time (60.1%). Caregivers were on average 46.3 years old (SD: 11.9), white (77.9%), caring for a parent with nmCRPC (47.7%), and were employed full‐time (73.2%). Additionally, the average age of the patients as reported by the caregivers was 65.5 years (SD: 14.0). The average disease duration since diagnosis for nmCRPC patients was approximately 4 years.

**Table 2:** Patient and Caregiver Demographic and Disease‐Related Characteristics

Characteristic	Patients (N = 143)	Caregivers (N = 149)
Age of patients, years		
Mean (SD)	53.04 (14.24)	65.50 (13.98)
Age of caregivers, years		
Mean (SD)	NA	46.32 (11.93)
Gender, n (%)		
Male	NA	60 (40.3)
Female	NA	89 (59.7)
Race, n (%)^a^, ^b^		
White	121 (84.6)	116 (77.9)
Black	16 (11.2)	21 (14.1)
Hispanic, n (%)		
Hispanic	16 (11.2)	22 (14.8)
Not Hispanic	127 (88.8)	127 (85.2)
Marital status, n (%)		
Single	26 (18.2)	32 (21.5)
Married	104 (72.7)	96 (64.4)
Divorced, separated or widowed	13 (9.1)	21 (14.1)
Education, n (%)^a^		
Less than college	21 (14.7)	37 (24.8)
College and above	120 (83.9)	112 (75.2)
Employment, n (%)^c^		
Employed full‐time	86 (60.1)	109 (73.2)
Employed part‐time	15 (10.5)	14 (9.4)
Homemaker	0 (0.0)	11 (7.4)
Retired	36 (25.2)	16 (10.7)
Unemployed	4 (2.8)	0 (0.0)
Disabled	3 (2.1)	2 (1.3)
Relation of patient to caregiver, n (%)^c^		
Parent	NA	71 (47.7)
Grandparent	NA	6 (4.0)
Spouse	NA	29 (19.5)
Sibling	NA	16 (10.7)
Relative	NA	15 (10.1)
Friend	NA	10 (6.7)
Duration of diagnosis, years		
Mean (SD)	4.25 (5.15)	3.72 (3.95)
Median (IQR)	2 (2.0 to 5.0)	3 (2.0 to 4.0)
Previous chemotherapy, n (%)		
Yes	50 (35.0)	40 (26.8)
No	93 (65.0)	109 (73.2)
Other cancer, n (%)		
Yes	8 (5.6)	8 (5.4)
No	135 (94.4)	141 (94.6)
Current medications for prostate cancer, n (%)^d^		
Leuprolide	49 (34.3)	48 (32.2)
Flutamide	30 (21.0)	20 (13.4)
Bicalutamide	24 (16.8)	27 (18.1)
Nilutamide	14 (9.8)	13 (8.7)
Goserelin	11 (7.7)	22 (14.8)
Enzalutamide	10 (7.0)	13 (8.7)
Abiraterone	6 (4.2)	14 (9.4)
Histrelin	5 (3.5)	13 (8.7)
Triptorelin	5 (3.5)	16 (10.7)
Apalutamide	3 (2.1)	6 (4.0)
Ketoconazole	3 (2.1)	5 (3.4)
No medications	9 (6.3)	5 (3.4)
Other medications	6 (4.2)	4 (2.7)
Don't know	4 (2.8)	8 (5.4)

Note

NA = not applicable.

^a^ Six (4.2%) and 2 (1.4%) patients reported “other” for race and education, respectively.

^b^ Eleven (7.4%) caregivers reported “other” for race and 1 (0.7%) caregiver declined to answer the question.

^c^ One (0.7%) and 2 caregivers (1.3%) reported “other” for employment and relation to patient, respectively. (Note: multiple sections were allowed for employment categories.)

^d^ Patients and caregivers were asked to report all the current medications that they/their patients were on.

### 3.2 Patient benefit‐risk preferences and relative importance of attributes

**Figure 2:**
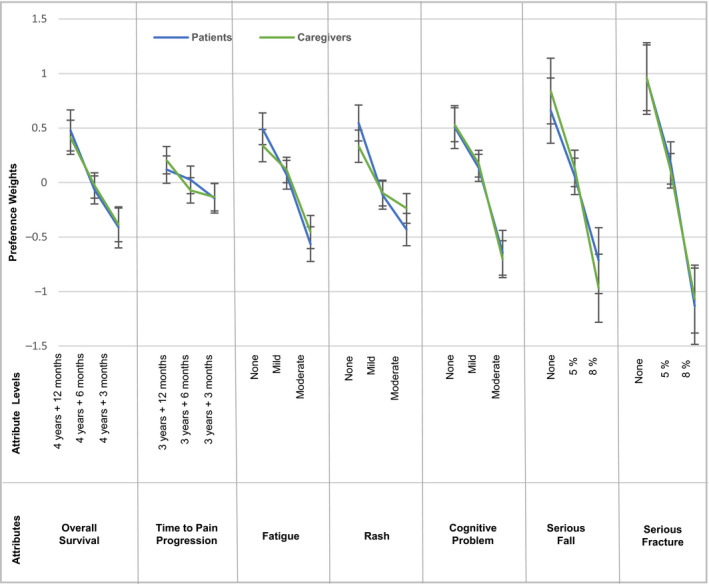
Estimated Preferences Weights of Patients and Caregivers. Vertical bars denote the 95% confidence intervals around estimates

**Figure 3:**
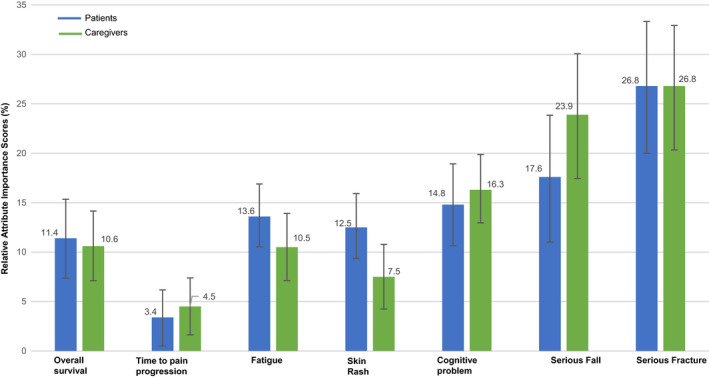
Relative Importance Scores of Treatment Attributes. Vertical bars denote the 95% confidence intervals around estimates

Figure 2 depicts the estimated preference weights from the RPL models. Both patients’ and caregivers’ preferences were logically ordered, that is, higher efficacy and lower risks were preferred over lower efficacy and higher risks. Figure 3 presents the relative importance of each attribute on patients’ and caregivers’ treatment choices. Of the two efficacy attributes, both patients and caregivers valued a 9‐month improvement in OS more than a similar improvement in TPP. Overall, both patients and caregivers viewed a reduction in AEs in the following descending order of importance: risk of a serious fracture from 8% to none, risk of a serious fall from 8% to none, moderate to no cognitive problems, moderate to no fatigue, and moderate to no skin rash. Among nmCRPC patients, reducing AEs was on average more important than improving OS across the levels assessed in the DCE. Although caregivers placed more importance on reducing serious fracture, serious fall, and cognitive problems, they placed similar importance on the reduction of fatigue and OS improvement, and relatively less importance on the reduction of skin rash compared to OS improvement.

### 3.3 Trade‐offs between overall survival and adverse events

The amount of OS that patients and caregivers were willing to forego in return for reduction in AEs are presented in Table 3. Patients and caregivers were willing to trade more months of OS for reduction in risks that they viewed as more important, that is, serious fracture, serious fall, and cognitive problems. For a reduction in cognitive problems from moderate to mild, nmCRPC patients were willing to trade 8.7 months of OS whereas caregivers were willing to trade more than 9 months of OS. This means that if the only difference between two treatment options was treatment A resulted in moderate cognitive problems and treatment B resulted in mild cognitive problems, in order for patients to view both treatments as equivalent, they would require 8.7 months of additional OS to compensate for the higher level of cognitive problems associated with treatment A. Further, the amount of OS that patients and caregivers were willing to trade to reduce cognitive problems from mild to none were 3.6 and 3.4 months, respectively. Of note, the range of OS levels assessed in the study was 9 months (from 4 years and an additional 3 months to 4 years and an additional 12 months); therefore, estimates beyond the range of 9 months were not calculated. By interpolating the estimates for none and 5% risks, it appeared that patients were willing to forego 5.8 and 4.0 months of OS to reduce the risk of serious fracture and serious fall, respectively, from 3% to none. The corresponding months of OS that caregivers were willing to forego were 6.6 and 5.4.

**Table 3:** Number of Months of Overall Survival that Patients and Caregivers Were Willing to Trade in Return for a Reduction in Adverse Events

	Patients		Caregivers	
	Reduction, Months of OS^a^	95% CI	Reduction, Months of OS^a^	**95% CI**
Fatigue				
Moderate to none	>9	‐	8.4	(5.0, 14.4)
Moderate to mild	7.0	(3.7, 13.0)	6.2	(3.4, 11.0)
Mild to none	5.5	(2.8, 9.8)	2.2	(−0.2, 5.1)
Skin rash				
Moderate to none	>9	‐	6.5	(3.4, 11.5)
Moderate to mild	3.3	(0.8, 7.1)	1.5	(−0.9, 4.3)
Mild to none	7.0	(3.8, 12.9)	5.0	(2.3, 9.3)
Cognitive problems				
Moderate to none	>9	‐	>9	‐
Moderate to mild	8.7	(5.1, 15.6)	>9	‐
Mild to none	3.6	(0.8, 7.5)	3.4	(0.8, 6.7)
Serious fall				
8% to none	>9	‐	>9	‐
8% to 5%	8.9	(4.2, 17.1)	>9	‐
5% to none	6.7	(2.2, 13.7)	9.0	(4.4, 16.1)
3% to none^b^	4.0	(1.3, 8.2)	5.4	(2.7, 9.7)
1% to none^b^	1.3	(0.4, 2.7)	1.8	(0.9, 3.2)
Serious fracture				
8% to none	>9	‐	>9	‐
8% to 5%	>9	‐	>9	‐
5% to none	>9	‐	>9	‐
3% to none^b^	5.8	(3.1, 8.5)	6.6	(4.1, 9.2)
1% to none^b^	1.9	(1.0, 2.8)	2.2	(1.4, 3.1)

Abbreviations: OS, overall survival.

95% CIs were estimated by simulating (10,000 draws) the multivariate normal distribution defined by the covariance matrix of the parameters from the random parameters logit models.

^a^ Range of OS levels beyond 9 months (ie what was measured in the study) was not estimated and was reported as “>9” in the table.

^b^ Improvements of risks from 3% to none and 1% to none were interpolated based on the estimates from 5% to none.

## 4 DISCUSSION

As SGARIs are emerging as new treatment options for nmCRPC patients given their impact on improving MFS and OS,^11^, ^12^, ^13^, ^14^, ^15^, ^16^ this study provides new and timely insights on how patients and caregivers weigh the benefits and risks of these new treatments. While these new SGARIs are similar in efficacy, they have different safety profiles that may differentially impact patients’ QoL. Furthermore, most nmCRPC patients are asymptomatic, thus avoidance of treatment‐related AEs is an important consideration. Our study is the first to systematically quantify the types of SGARI‐related AEs that are most important to patients and caregivers and the extent of treatment‐related benefits (ie months of OS) they are willing to forego in order to reduce the risks associated with these new treatments.

Our study results demonstrate that treatment‐related AEs had a great influence on patients’ and caregivers’ treatment choices. In fact, among our study respondents, avoiding an 8% risk of serious fracture, 8% risk of serious fall, and moderate cognitive problems had a greater influence on treatment choices compared to extending OS by 9 months (from 4 years and 3 months). Both patients and caregivers viewed AEs in the following order of importance (most to least): serious fracture, serious fall, cognitive problems, fatigue, and rash. These findings were similar to the information elicited from the qualitative and pre‐test interviews, in which patients and caregivers often expressed that they were more concerned with avoiding treatment‐related risks rather than extending benefits in terms of OS or delaying pain progression. Additionally, a similar study conducted among nmCRPC‐treating physicians demonstrated that among the AE attributes, physicians were most concerned with cognitive problems and serious fracture while making treatment choices, and this aligns with the findings from the patients and caregivers.^38^ Understanding patient preferences can also help to facilitate clinical treatment decision‐making, and it has been shown that the optimal treatment for prostate cancer depends on a combination of clinical scenarios (eg patient age and tumor aggressiveness) and the patient's preferences.^39^

Our results also show that patients and caregivers were willing to trade significant amounts of OS to avoid treatment‐related risks and were willing to trade a higher amount of OS to avoid risks that they viewed as more important. Other studies have also observed that cancer patients were willing to make substantial trade‐offs between survival and AEs. For example, in castration‐resistant prostate cancer or biochemically recurrent prostate cancer, fatigue was found to be an important risk attribute for patients, and one study observed that these patients valued reducing fatigue more than improving OS.^25^, ^40^ In another study, patients with advanced non‐small cell lung cancer valued a reduction in fatigue severity from moderate to none equally as an additional 7.3 months of progression‐free survival (with mild disease symptoms).^41^ Taken together, these findings underscore the importance of carefully balancing the goal of improving survival with the introduction of treatment‐related AEs.

This study has some limitations, one of which is typical of DCEs, in that respondents’ preferences between hypothetical profiles may not be truly reflective of their actual treatment choices. Our study was also not powered to detect differences between the patient and caregiver subgroups and comparisons should only be drawn qualitatively. Sample size estimation in DCEs is known to be challenging; while we followed rule‐of‐thumb recommendations, a larger sample size would have provided smaller CIs to allow for comparisons. The use of online panels for recruitment may have limited the generalizability of the results to the wider nmCRPC population, as patients and caregivers who participate in online panels may have different characteristics than those who do not (eg younger, more educated, greater access to technology), which could affect their benefit‐risk trade‐offs. We did observe a relatively young sample of patients in this study, as expected from the use of online panels. Similar DCE studies in oncology that utilized online panels also reported study populations with relatively young mean ages (<55 years old).^42^, ^43^ We further explored this by comparing the RAISs between younger and older patients in our sample. Some differences in the importance ranking of attributes did change with respect to fatigue, rash, and cognitive problems, but the top two most important and two least important attributes remained the same (results not shown). Lastly, while we conducted a targeted literature review and qualitative interviews from all perspectives, including patients, caregivers, and physicians, to ensure that salient attributes were included in the DCE, we cannot rule out the possibility of omitted variable bias, where a relevant attribute that can affect treatment choices is not included in our assessment. One possible attribute is the cost of treatment. However, the focus of this study was to look at trade‐offs between AEs and OS and their relative importance in treatment choices, instead of willingness‐to‐pay. Nonetheless, future research should explore the extent to which costs can influence treatment choices in this population.

## 5 CONCLUSIONS

The treatment landscape for nmCRPC is changing with the recent emergence and approval of SGARIs. Unlike first generation AR inhibitors, SGARIs demonstrated a significant clinical benefit through the prolongation of MFS and in particular OS in this patient population.^11^, ^12^, ^13^, ^14^, ^15^, ^16^ However, since this patient population is relatively asymptomatic, it is important to carefully balance risks and benefits when selecting SGARI treatments to optimize the quality of a nmCRPC patient's survival. Our results indicate that nmCRPC patients and caregivers preferred treatments with lower AE burden and were willing to forego substantial amounts of OS to reduce the risk and severity of AEs. Among the AEs evaluated, they valued the reduction of serious fracture, serious fall, and cognitive problems, the most.

## CONFLICT OF INTEREST

SS consulted for Bayer US LLC and Janssen, and has received research funding from Bayer and Pfizer. AFM and ARW are full‐time employees of Bayer US LLC and own stocks in Bayer. SJS is a full‐time employee of Bayer US LLC and own stocks in Bayer and Pfizer. SA is a full‐time employee of Bayer US LLC. MB is a shareholder of Pharmerit International, the institution which received funding from Bayer, for conducting the study. MB, XN, NJ, JT, and JF are full‐time employees of Pharmerit International.

## AUTHORS CONTRIBUTIONS

SS, AFM, SA, MB, ARW, and SJS contributed to the study design, results interpretation and manuscript writing. XN, NJ, and JT contributed to the study design, data collection, data analysis, results interpretation, and manuscript writing. JF contributed to the data collection, results interpretation, and manuscript writing.

## Supporting information

Supplementary Material

## Data Availability

The datasets during and/or analyzed during the current study available from the corresponding author on reasonable request.
