# Supplementary material for: Patient and caregiver benefit‐risk preferences for nonmetastatic castration‐resistant prostate cancer treatment
Source: Cancer Med. 2020 Jul 29;9(18):6586–96. doi: 10.1002/cam4.3321 (PMC7520320; doi:10.1002/cam4.3321)
Supplement: Supplementary file 1 — Supplementary Material [file CAM4-9-6586-s001.docx]

**Appendix**

**Table 1 Labels and Descriptions Used for Efficacy Attributes and Levels**

| **Attribute Label** | **Attribute Description** | **Attribute Level Description** | | |
| --- | --- | --- | --- | --- |
| Prolonging life | For this survey, assume that people with this type of prostate cancer usually live for another 4 years. The medicine may help to slow or stop the growth of the prostate cancer such that people live a few months longer (for example, 4 years and an additional ___ months). | 4 years and an additional 12 months  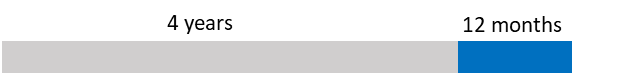 | 4 years and an additional 6 months  **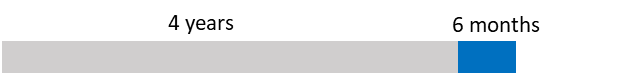** | 4 years and an additional 3 months  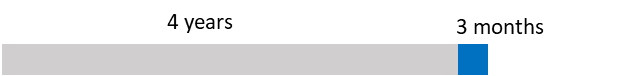 |
| Delay in time until pain progresses (develops or worsens) | For this survey, assume that people with this type of prostate cancer usually have 3 years until their pain progresses. People with this type of prostate cancer may experience a progression of pain over time. This means that people develop pain if they are not currently experiencing pain, or they will experience pain that is worse than what they currently have. The progression of pain bothers them, and the doctor thinks that this is important to address.  The medicine can better control the cancer and delay the time until the pain starts to progress (develop or worsen) by a few more months (for example, 3 years and an additional ____months). | 3 years and an additional 12 months  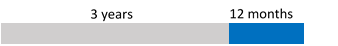 | 3 years and an additional 6 months  **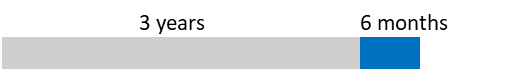** | 3 years and an additional 3 months  **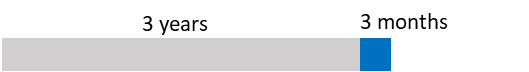** |

**Table 2 Labels and Descriptions Used for Risk (Adverse Event) Attributes and Levels**

| **Attribute Labels** | **Attribute Description** | **Attribute Level Description** | | |
| --- | --- | --- | --- | --- |
|  |  | **None** | **Mild** | **Moderate** |
| Fatigue (lack of energy) | People who take the medicine may experience fatigue as a side effect. This means that they feel weak and have a lack of energy to do their daily activities. | No fatigue. | **(does not affect daily activities)**: Fatigue goes away with rest and does not limit your daily activities. | **(affects daily activities):** Fatigue does not go away with rest and limits your daily activities such as shopping for groceries or doing housework. |
| Skin rash | People who take the medicine may develop a skin rash as a side effect. Symptoms of rash can include itchiness, a burning sensation, feeling of tightness, feeling of tenderness, or blisters. | No skin rash. | **(less than 10% of the body, does not affect daily activities):** Skin rash that affects only a small area of the body (less than 10%) and does not limit your daily activities. | **(10-30% of the body, affects daily activities):** Skin rash that is limited to certain areas of the body (10-30% of the entire body). Because of the rash, you may avoid social activities. The skin rash limits your daily activities such as shopping for groceries and doing housework. |
| Cognitive problems | People who take the medicine may develop cognitive problems as a side effect. These problems can include being confused, having trouble remembering things, concentrating, learning new things, thinking clearly, and making decisions in everyday life. | No cognitive problems. | **(does not affect daily activities):** You begin to have some problems with confusion, remembering things, concentrating, learning new things, thinking clearly, or making decisions. However, these problems are minor and do not affect your work responsibilities and your daily activities. | **(affects daily activities):** You have some problems with confusion, remembering things, concentrating, learning new things, thinking clearly, or making decisions. You face challenges keeping up with your work responsibilities and daily activities such as managing money, or shopping for groceries. |
| **Attribute** | **Attribute Description** | **None** | **5%** | **8%** |
| Chance of a serious fall | People who take the medicine may have a higher chance of experiencing a serious fall as a side effect. A serious fall results in injuries that require people to be admitted to a hospital. | None (0 out of 100 people)  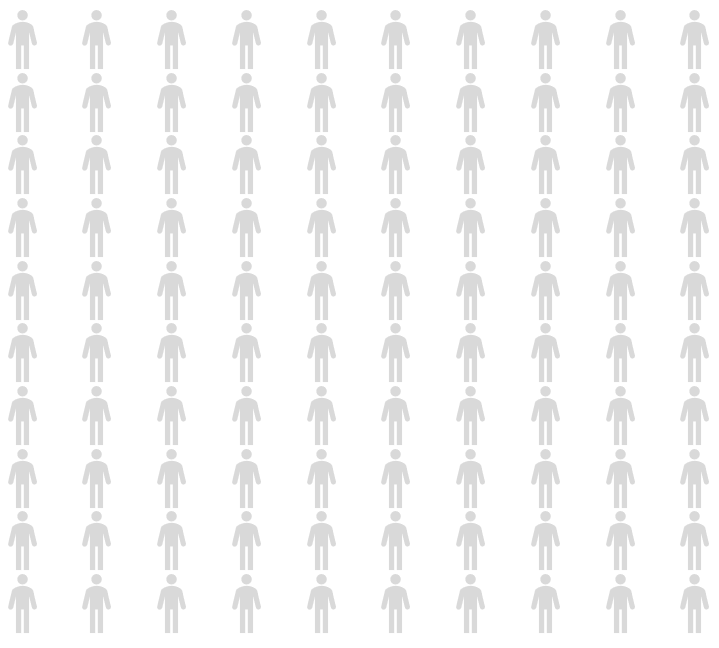 | 5% (5 out of 100 people) 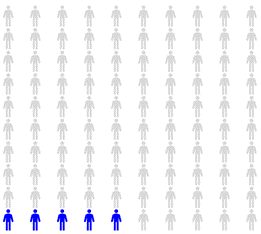 | 8% (8 out of 100 people)  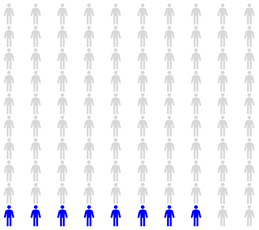 |
| Chance of a serious fracture | People who take the medicine may have a higher chance of experiencing a serious fracture (broken bone) as a side effect. A serious fracture is disabling (severely limits movement and/or activities, and which may be permanent). The bone may be dislocated (moved from its original location) and may be visible. Immediate medical care or surgery is needed to fix the serious fracture. This may be life-threatening. | None (0 out of 100 people)  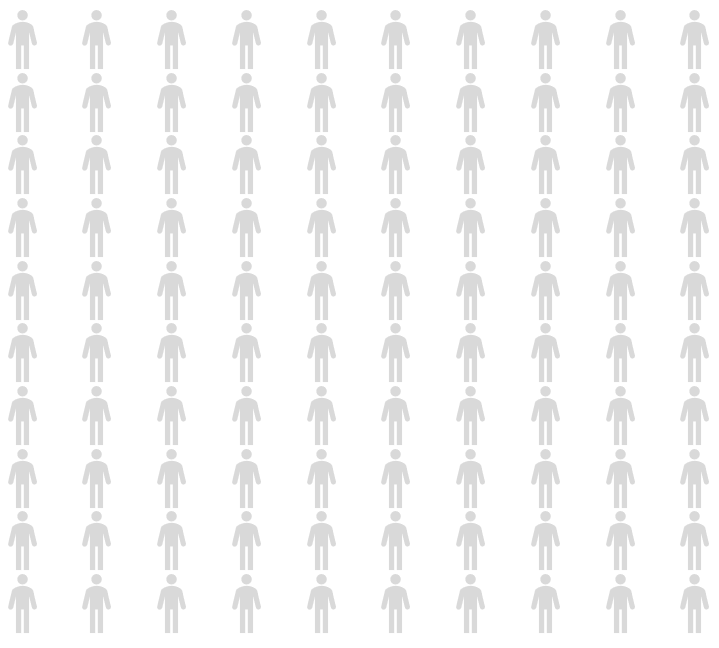 | 5% (5 out of 100 people)  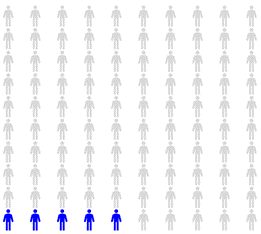 | 8% (8 out of 100 people)  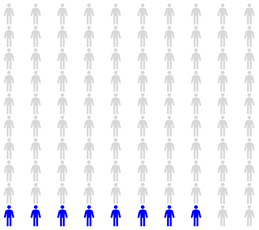 |

**Risk Grids Used in Survey**

To explain the risks probabilities used to describe the levels for the serious fall and serious fracture attributes, we included the following explanation and exercise:

| 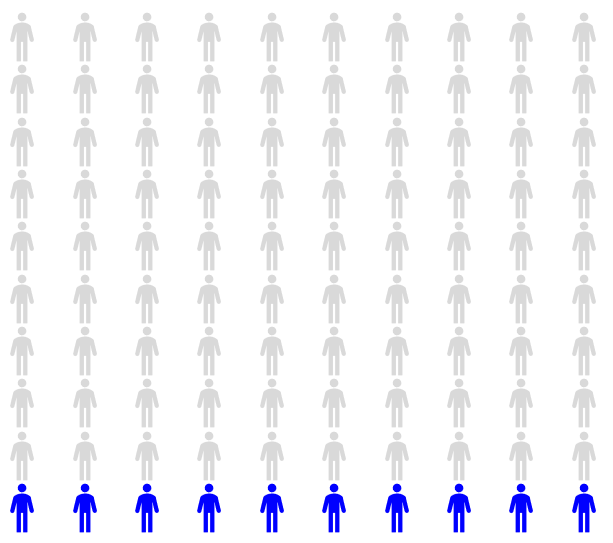 | In the picture on the left, every person in the picture represents 1 person who is taking a medicine for their prostate cancer. There are 100 people in the picture. The people in blue represent the number of people that will experience a **serious fall**. The people in grey represent the number of people that will not experience a **serious fall**.  In this example, there are 10 people in blue color. That means that 10 people out of 100 (10%) will experience a **serious fall** as a side effect of the medicine. |
| --- | --- |

Now, let’s try an example:

| 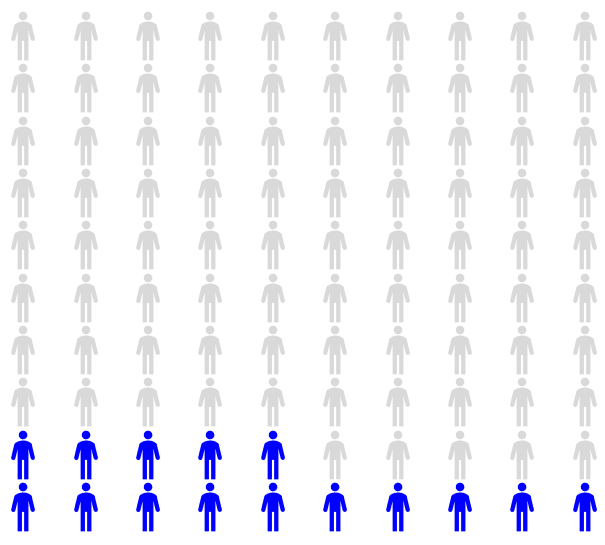 | Please look at the picture to the left.  Every person (blue or grey) in the picture represents 1 person who is taking a medicine for their prostate cancer. There are 100 people in the picture. The people in blue represent the people that will experience a **serious fall** as a side effect of the medicine.  In this example, what is the chance of experiencing a **serious fall** as a side effect of the medicine?   - 5 out of 100 people (5%) - 15 out of 100 people (15%) - 85 out of 100 people (85%) - 95 out of 100 people (95%) |
| --- | --- |

If the incorrect answer is chosen, a pop-up will appear with the following explanation: “Remember, each blue person represents a person who takes the medicine for prostate cancer and will have a serious fall as a side effect. There are 15 blue people, so 15 out of 100 (15%) people will have a serious fall.”

Responses to the above exercise showed that 19.6% and 7.7% of patients incorrectly answered the exercise for serious fall and serious fracture, respectively. The proportions of caregivers who incorrectly answered the exercise for serious fall and serious fracture were 12.1% and 9.4%, respectively. Since the exercise for serious fall was shown before serious fracture, and the answers to both exercises were the same, we would expect a decrease in the proportions of respondents who gave incorrect answers to the second exercise (i.e., serious fracture) and this was indeed observed. With each incorrect answer, a pop-up message with the explanation (see above) and correct answer was provided to improve respondents’ comprehension of the risk grids.

**Figure 1 Screening Attrition Flowchart**


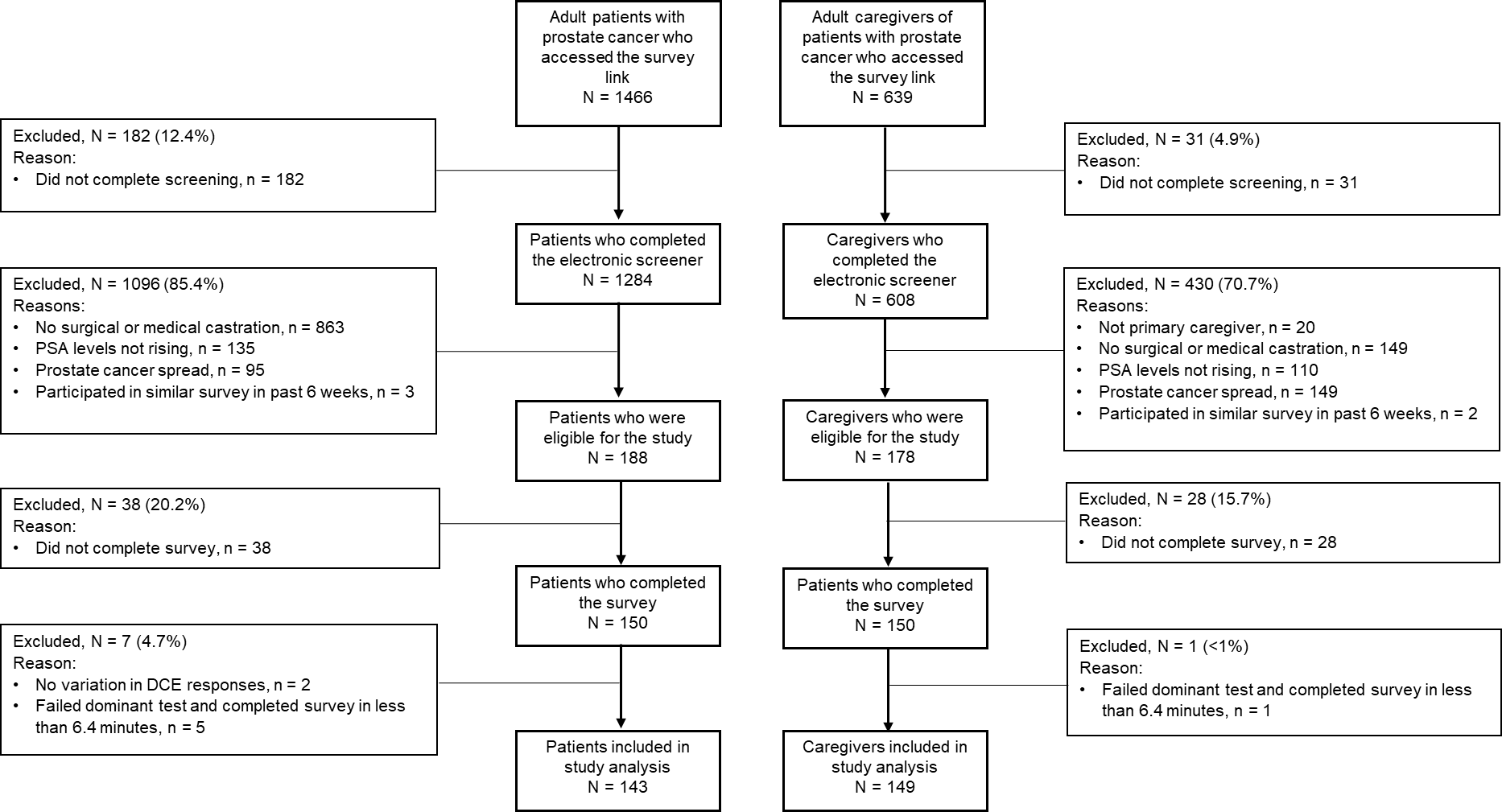


**Online screening tool for nmCRPC**

1. What is your age?

- Less than 18 years [STOP, INELIGIBLE]
- 18 – 44 years [CONTINUE, GO TO Q2]
- 45 – 64 years [CONTINUE, GO TO Q2]
- 65 years and over [CONTINUE, GO TO Q2]

1. Have you ever been diagnosed by a physician with any of the following conditions? Please check all that apply.

- Bladder cancer
- Colon cancer
- Kidney cancer
- Lung cancer
- Leukemia
- Lymphoma
- Prostate cancer [CONTINUE, GO TO Q4]
- Skin cancer
- Thyroid cancer
- None of the above [CONTINUE, GO TO Q3]

[If ≥ 5 cancers are selected, STOP, INELIGIBLE]

1. Are you the caregiver of a patient (≥ 18 years old) who has any of the following conditions? Please check all that apply.

- Bladder cancer
- Colon cancer
- Kidney cancer
- Lung cancer
- Leukemia
- Lymphoma
- Prostate cancer [CONTINUE, GO TO Q7]
- Skin cancer
- Thyroid cancer
- None of the above [STOP, INELIGIBLE]

[If ≥ 5 cancers are selected, STOP, INELIGIBLE]

**AT THIS POINT, THE SCREENER WILL BREAK OUT INTO 2 SECTIONS – ONE FOR PATIENT, AND ONE FOR CAREGIVER - PATIENTS WILL SEE Q4 TO Q7; CAREGIVERS WILL SEE Q8 TO Q12

*****PATIENT SCREENER*****

1. From this point forward, we will be asking you questions related to your prostate cancer.

Which of the following treatments/procedures have you undergone for your prostate cancer? Please check all that apply.

- I had surgery to remove both testicles (bilateral orchiectomy) [CONTINUE, GO TO Q5]
- I am taking medicine to control my hormonal levels (for example, Eligard®/Lupron®, Supprelin®/Vantas®, Trelstar ®, Zoladex ®) [CONTINUE, GO TO Q5]
- None of the above [STOP, INELIGIBLE]

1. Have you been told by a physician that your prostate-specific antigen (PSA) levels are rising?

- Yes [CONTINUE, GO TO Q6]
- No [STOP, INELIGIBLE]
- I do not know [STOP, INELIGIBLE]

1. Have you been told by a physician that your prostate cancer has spread to any other part of your body?

- Yes [STOP, INELIGIBLE]
- No [CONTINUE, GO TO Q7]

1. Have you participated in an online survey about non-metastatic castration-resistant prostate cancer in the last 6 weeks?

- Yes [STOP, INELIGIBLE]
- No [ELIGIBLE, CONTINUE TO SURVEY]

*****CAREGIVER SCREENER*****

1. From this point forward, we will be asking you questions relating to the patient with prostate cancer that you are caring for. Please answer the question based on what you know about his prostate cancer.

Are you the primary caregiver of the patient with prostate cancer?

(The primary caregiver is someone who is most often available to assist or support the patient with prostate cancer, and does not receive payment in return for providing support.)

- Yes [CONTINUE, GO TO Q9]
- No [STOP, INELIGIBLE]

1. Which of the following treatments/procedures has the patient undergone for his prostate cancer? Please check all that apply.

- He had surgery to remove both testicles (bilateral orchiectomy) [CONTINUE, GO TO Q10]
- He is taking medicine to control his hormonal levels (for example, Eligard®/Lupron®, Supprelin®/Vantas®, Trelstar ®, Zoladex ®) [CONTINUE, GO TO Q10]
- None of the above [STOP, INELIGIBLE]

1. Has the patient been told by a physician that his prostate-specific antigen (PSA) levels are rising?

- Yes [CONTINUE, GO TO Q11]
- No [STOP, INELIGIBLE]
- I do not know [STOP, INELIGIBLE]

1. Has the patient been told by a physician that his prostate cancer has spread to any other part of his body?

- Yes [STOP, INELIGIBLE]
- No **[**CONTINUE, GO TO Q12**]**

1. Have you participated in an online survey about non-metastatic castration-resistant prostate cancer in the last 6 weeks?

- Yes [STOP, INELIGIBLE]
- No **[ELIGIBLE, CONTINUE TO SURVEY]**
